# The Function of Transthyretin Complexes with Metallothionein in Alzheimer’s Disease

**DOI:** 10.3390/ijms21239003

**Published:** 2020-11-26

**Authors:** Natalia Zaręba, Marta Kepinska

**Affiliations:** Department of Biomedical and Environmental Analysis, Faculty of Pharmacy, Wroclaw Medical University, Borowska 211, 50-556 Wroclaw, Poland; natalia.zareba@umed.wroc.pl

**Keywords:** Alzheimer’s disease, β-amyloid, metallothionein, protein-protein interaction, transthyretin

## Abstract

Alzheimer’s disease (AD) is one of the most frequently diagnosed types of dementia in the elderly. An important pathological feature in AD is the aggregation and deposition of the β-amyloid (Aβ) in extracellular plaques. Transthyretin (TTR) can cleave Aβ, resulting in the formation of short peptides with less activity of amyloid plaques formation, as well as being able to degrade Aβ peptides that have already been aggregated. In the presence of TTR, Aβ aggregation decreases and toxicity of Aβ is abolished. This may prevent amyloidosis but the malfunction of this process leads to the development of AD. In the context of Aβplaque formation in AD, we discuss metallothionein (MT) interaction with TTR, the effects of which depend on the type of MT isoform. In the brains of patients with AD, the loss of MT-3 occurs. On the contrary, MT-1/2 level has been consistently reported to be increased. Through interaction with TTR, MT-2 reduces the ability of TTR to bind to Aβ, while MT-3 causes the opposite effect. It increases TTR-Aβ binding, providing inhibition of Aβ aggregation. The protective effect, assigned to MT-3 against the deposition of Aβ, relies also on this mechanism. Additionally, both Zn_7_MT-2 and Zn_7_MT-3, decrease Aβ neurotoxicity in cultured cortical neurons probably because of a metal swap between Zn_7_MT and Cu(II)Aβ. Understanding the molecular mechanism of metals transfer between MT and other proteins as well as cognition of the significance of TTR interaction with different MT isoforms can help in AD treatment and prevention.

## 1. Introduction

**Figure 1:**
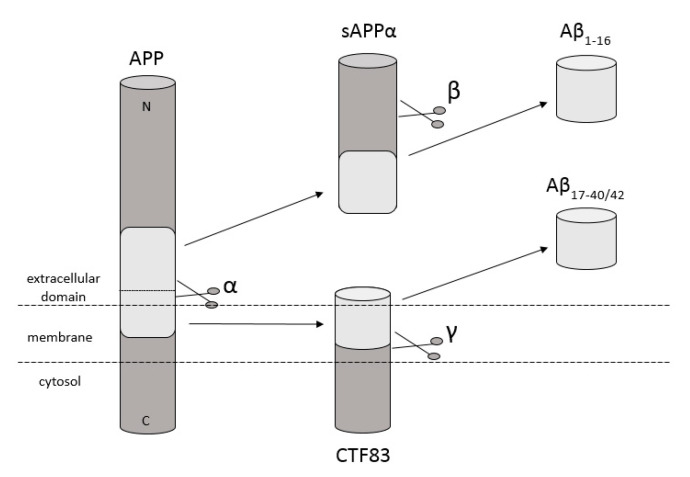
Schematic diagram illustrating proteolytic cleavage of the APP in the non-amyloidogenic pathway, when APP is cleaved by α- and γ-secretase, respectively, and forms Aβ_17–40/42_, or when APP is cleaved by α- and β-secretase and forms Aβ_1–16_ peptides. Based on [4].

**Figure 2:**
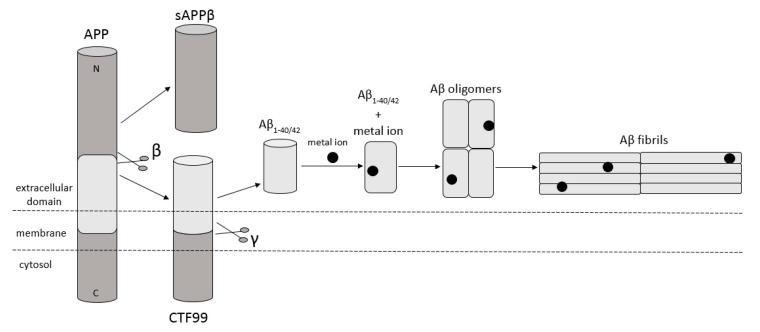
Schematic diagram illustrating cleavage of APP in the amyloidogenic pathway, when APP is cleaved by β- and γ-secretase, respectively, and forms full-length Aβ_1–40/42_ peptides. This peptide can interact with metal ions from the brain, forming Aβ oligomers and then Aβ fibrils. Based on [4].

**Figure 3:**
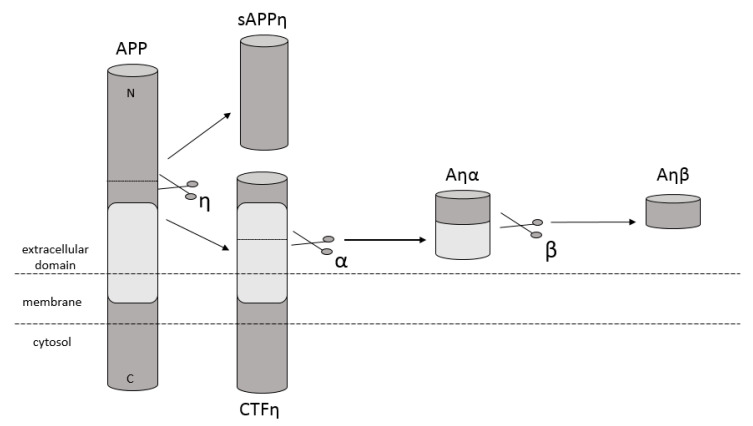
Schematic diagram illustrating proteolytic cleavage of APP in the ƞ-secretase pathway, when APP is cleaved by ƞ-secretase and then by α- and β-secretase, respectively, and forms Aƞα and Aƞβ peptides accordingly.

Amyloidosis is a disease in which pathological extracellular fibers, being filamentary structures formed by insoluble protein, build up in tissues and organs. Alzheimer’s disease (AD) is one of the local amyloidoses in which amyloid-β peptide (Aβ) is deposited. The disease is characterized by the existence of intracellular neurofibrillary tangles (NFTs) resulting from the accumulation of tau protein associated with microtubules as well as the presence of extracellular senile plaques [1]. These changes occur in the central nervous system (CNS) and, in particular, in the hippocampus and neocortex. Senile plaques are composed of transition metal ions such as Cu^2+^ or Zn^2+^ at high concentrations [2] and, mainly, conformationally changed, aggregated, and protease-resistant Aβ [2,3]. The peptide comprises 40 to 43 amino acids and is formed from the amyloid precursor protein (APP) through the proteolytic action of β- and γ-secretase. APP is a transmembrane protein expressed particularly in the CNS [4]. Aβ peptide is able to rise through three different pathways of APP metabolism. The first and principal pathway, namely the non-amyloidogenic pathway (Figure 1), assumes cleavage of APP at first by α-secretase between the 612 and 613 amino acids [5]. It leads to the emergence of APPα and a membrane-associated carboxy-terminal fragment (CTF83). Subsequently, γ-secretase forms Aβ_17–40/42_ peptides or β-secretase generates Aβ_1–16_ peptide. The other pathway is the amyloidogenic pathway (Figure 2). It is less common and involves the cleavage of APP at the beginning by β-secretase, leading to the emergence of APPβ and a membrane-associated carboxy-terminal fragment (CTF99). Subsequently, γ-secretase generates mainly full-length Aβ_1–40/42_ peptide, which could subsequently affect metal ions in the brain, form soluble oligomers and fibrils, and thus creating senile plaques (Figure 2) [4,6]. The third pathway of APP metabolism that has been recently discovered involves η-secretase that cleaves APP at the 504 to 505 amino acids and generates Aηα and Aηβ, which are lower molecular mass carboxy-terminal fragments following second cleavage by α- and β-secretase, respectively (Figure 3) [4,7]. The first form (Aƞα) includes Aβ_1–16_ peptide in its sequence, which is often regarded as neurotoxic [4]. Full-length peptides, however, are the most abundant isoforms that are toxic to cells and could lead to cellular death [4,6,8]. Their accumulation in the form of aggregates indicates the development of the disease [9]. For neuronal cells, toxic effects of Aβ can also be triggered by other actions, such as internalization via pinocytosis, endocytosis and phagocytosis [10], ion pore formation, and interaction with the serpin-enzyme complex receptor. Aβ can also be a receptor of advanced glycation end products and oxidative stress damage [11].

Due to the fact that Aβ peptides are produced in the brain mainly during normal neuronal activity and are also present in soluble form in the blood and cerebrospinal fluid (CSF) [4,12], a potentially advantageous method of controlling the physiological levels of Aβ could be the reduction of amyloid plaques accumulation and inhibition of AD progression rather than a total inhibition of the peptide [8,13]. Generally, the control is possible thanks to adjusting the balance between the production of Aβ and its degradation [13]. However, in recent years, only a few anti-amyloid agents have shown potential therapeutic benefits at the clinical trials stage. Aducanumab, gantenerumab, BAN2401, and ALZ-801 should be mentioned [14]. The first three are injectable human anti-Aβ antibodies, which promote the removal of this peptide. The fourth one is an oral drug, which preferentially inhibits oligomer formation, excluding plaque binding [14]. The highest possible doses of aducanumab and BAN2401 show low efficacy, and dose increases are limited by the possible occurrence of vasogenic edema. Selective AZ-801, the use of which is not associated with the previously mentioned risk of vasogenic edema, allows these limitations to be bypassed [14]. Perhaps in the near future we will see the approval of the first drugs for AD. However, none of the listed candidates involve metal chelation as a target or an intermediate target. Yet the theory of metal chelation as an effective pathway to successful AD treatment has been analyzed, and despite evidence of its ineffectiveness, research is still ongoing in this direction [15]. The most recent studies involve multitarget-directed ligands (MTDLs) that are designed to function on multiple AD targets [16,17,18] or metal protein attenuating compounds (MPACs) capable of crossing the BBB, that normalize the dyshomeostasis of metal concentrations by competing with the target protein [17]. If late-stage agents are approved by the FDA, the drugs are expected in three to five years. Conversely, if potential candidates fail the approval procedure, alternative ways, potentially leading to effective treatment of AD, will be analyzed.

## 2. Significance of Metals Ions in Alzheimer’s Disease

The brain is one of the organs with the highest content of d-block metal ions such as Zn, Cu, Fe, Co, Cr, Mo, and Mn per weight unit [4]. As mentioned above, metal ions that are present in the brain also play an important role in the process of Alzheimer’s pathogenesis, mainly due to the existing evidence for metal homeostasis disorders in AD patients. It especially concerns Cu, Zn, and Fe ions [4]. Cu, Fe, and other metals such as Zn, are highly reactive metals, thus they have to be strictly controlled by intra- and extracellular transporters and binding proteins [19]. In a healthy brain, they play a role mainly in metalloproteins as an electron transfer site, catalytic center, or structural component [4]. Similarly to many other neurological diseases, during AD there is an imbalance in the blood–brain barrier (BBB) action that is probably closely related to the disturbance of metals homeostasis [6]. The BBB is a diffusion border with high selectivity, which isolates the blood circulation from the brain interstitial fluid and is necessary for the proper functioning of the CNS [20]. In a properly functioning CNS, defense functions are performed by astrocytes which are the elements forming the BBB [20], and microglia, which are resident immune cells of the CNS [21]. During the occurrence of pathological conditions, the same structures can increase inflammation and take part in cellular damage [20]. Interaction of microglia, astrocytes, and the immune system causes changes in the production of neurotoxins and neurotrophins by these cells, which leads to neuronal damage and synaptic dysfunction. As a result, a breakdown of the BBB occurs, which is a factor in the pathogenesis of AD [20,22]. The cascading events occur through the action of β-amyloid protein and related oligopeptides which activate microglia and astrocytes [20]. In the CNS, processes of absorption, distribution, biotransformation, and excretion take place in the brain barrier systems, including the BBB. During those processes, biological mechanisms regulating Fe and Cu homeostasis, inter alia, are activated. Pathological BBB circumstances of functional or structural character can cause homeostasis disorders related to metal demand and supply in the CNS [19].

The Cu^2+^ and Zn^2+^ ions are concentrated within senile plaques of patients with Alzheimer’s disease. Those plaques are directly bound to Aβ that has selective affinity binding sites for those ions [6,23]. Cu^2+^ ions have stronger affinity than Zn^2+^ [23] and in acidic conditions, at pH 6.6, copper ions utterly supplant the ions of zinc [6]. Furthermore, Cu^2+^ binding significantly accelerates the rate of fiber formation and enhances cytotoxicity in cell culture [24]. Thereby, as a result of the coordination of redox-active metal ions, such as copper, protein accumulation might be influenced by the metal-catalyzed chemical modification of the protein. For example, reactive oxygen species (ROS) have been shown to generate an Aβ dimer by covalent cross-linking of tyrosine residues within Aβ [25]. Copper ions arrested in Aβ fibrils, which are electrochemically active and form ROS, give rise to oxidative stress and cytotoxicity. According to some pieces of evidence, it was proposed that the toxicity of amyloid aggregates depends on copper content [26]. However, the issue of the Cu transport mechanism inside and outside the brain is still not fully examined. It is known that during proper brain activity, Cu needs a special transport system that allows its movement through the brain barriers. Nevertheless, under certain pathological circumstances, Cu can pass through via diffusion because of abnormalities in the brain barrier permeability. This may cause the breakdown of the mechanisms responsible for Cu homeostasis, thus resulting in the development of neurodegenerative diseases including AD [19]. In contrast, zinc ions are redox-inert and have protective properties in AD. H_2_O_2_ from Aβ, created with the participation of Cu^2+^, is suppressed by Zn^2+^ [26]. These observations are supported by in vivo studies in animal models of AD that implicate Cu^2+^ impaired homeostasis in the promotion of the disease [27,28]. For instance, a rabbit model of Alzheimer’s disease showed that rabbits fed with copper in a high cholesterol diet developed amyloid plaques and learning deficits [28]. By contrast, transgenic mice presented a reduced AD pathology with increased intracellular copper levels [29,30]. What is interesting, Aβ from rats and mice had a different amino acid sequence, compared with humans, with the ability to reduce the interaction with metals ions. Thanks to this, these animals are the only mammals without cerebral Aβ amyloid accumulation with aging [6,23].

Metal ion coordination is a process that usually affects the net charge of a protein by adding the positive charge from the metal ion or subtracting it through repeated deprotonation. As a result of this process, a protein with acidic pH may become more neutrally charged, and at the same time more susceptible to self-association. This mechanism was proposed in the case of accelerating the formation of fibers by Cu^2+^ ions, which at pH 7.4 caused Aβ to approach the isoelectric point [31]. In addition, it seems that AD may be characterized by an increase in the unstable extracellular pool of Cu^2+^ ions, as well as intra-neuronal Cu^+^ ions, also binding to Aβ, which may therefore affect fiber formation [32,33]. Understanding the relationship between intra- and extracellular copper and its effect on Aβ is one of the key aspects for a better understanding of copper-related AD pathology.

Zn^2+^ ions concentrated in synaptic vesicles are assigned the role of regulating normal cognitive functions. The appropriate level of these ions is regulated by a special zinc transporter ZnT-3 [34], however, interestingly, the mouse model of AD with ZnT-3 knocked out does not develop amyloid plaques in the brain [35]. Coordination of the metal ions might also lead to cross-linking between molecules, and thus stabilization of fibrils or oligomers, which will affect misfolding and protein accumulation. For example, in vitro Zn^2+^ can form an inter-molecular complex with Aβ, crosslinking between histidine residues on multiple Aβ molecules, which inhibits fibrillization [36,37]. Otherwise, protein misfolding may become more energetically beneficial when the coordination of metal ions would destabilize the non-pathogenic structure.

It is evidenced that transition metal homeostasis in the brain is directly linked to AD development, and the concentration of Cu and Zn ions in amyloid fibers raises the possibility of triggering or promoting amyloid formation. However, as mentioned earlier, therapy based on classic chelation seems to be insufficient, however, restoring metal homeostasis as a target or one of the targets in the treatment of AD is not ruled out [16,17]. Therefore, the search for new strategies in the fight against AD is conducted.

## 3. Metallothioneins

Metallothioneins (MTs) are low molecular weight proteins, which in the case of humans have a single chain including 61 to 68 amino-acids, where thanks to the Cys sulfur atoms (Me-SCys), 20 cysteines residues bound 7 ions of divalent metals in total [38,39]. In general, metal binding by MT is directly linked to thiol groups derived from cysteine residues [38]. Nevertheless, sometimes it is associated with the possession of histidines and a nitrogen lateral chain [40,41]. Between the 31 and 32 amino acids, there is a boundary of MTs α- and the more reactive β-domain [42]. Both secondary and tertiary structures depend on the presence of metal ions: functional secondary structure appears after binding to metals and tertiary structure of these proteins depends on the amount of added metal ions and their nature [39]. MTs may occur in the cell as apotioneins (apo-MT, thioneins), which are not bound to metals [43]. These proteins are involved in the transport, storage, and concentration regulation of essential metal ions such as Zn and Cu, detoxification of heavy metals taking part in the maintenance of the intracellular redox balance, apoptosis, anti-inflammatory processes, and protection against free radicals and neuronal lesions [38,44,45,46]. At first, MTs were classified as intracellular proteins, and they were mainly located in the cytoplasm and nucleus by translocation. They could also be found in the mitochondria and lysosome. On the other hand, current findings confirm that MTs are also extracellularly active proteins occurring in plasma, amniotic and pancreatic fluid, and urine or milk [38,47]. Mammalian MT can be subdivided into four distinct isoforms: MT-1, -2, -3, and -4. The differences between them are mainly due to changes in the amino acid sequence [48]. MT-1 and MT-2 are the closest paralogs that differ only by one amino acid. In general, they have the same function, so they are commonly grouped together and referred to as MT-1/2 [49]. On the other hand, MT-3 contains additional threonine-elements in the N-terminal part and acidic hexapeptide in the C-terminal region. Additionally, MT-3 contains a Cys (6)-Pro-Cys-Pro (9) motif, which does not contain other MTs [47]. MT-4 contains Glu at position five compared to MT-1/2, and has 62 amino acids in total [42]. The physiological role of MT-3 does not only appear to differ from that of MT-1/2, but it also depends on the brain area [50,51] and on its putative partners [52]. These differences between MT isoforms may be crucial for the role they play.

## 4. Metallothioneins Expression in Alzheimer’s Disease

Regulation of MTs expression can take place at the transcriptional, post-transcriptional, translational, and post-translational levels. Additionally, few eukaryote examples also use epigenetic mechanisms [39]. Expression of MT can be induced by several kinds of stress and molecular signals. There are specific transcriptional regulation patterns within the cell which are probably associated with internal changes in metal concentration [39,53]. Astrocyte is the main type of cell that expresses MT isoforms, which is characterized by lower levels of expression in ependymal, epithelial cells of choroid plexus, meningeal cells of the pia mater, and endothelial cells of blood vessels. Neurons synthesize MT-1/MT-2 but to a considerably lesser extent than astrocytes [54]. Different patterns of expression suggest specific in vivo functions of each isoform [55]. An example is both MT-1 and -2, found in the liver, kidneys, and intestines, and is also present throughout the brain and spinal cord [38,54,56,57]. These two isoforms of MTs are primary zinc-binding proteins, and when they are exposed to heavy metals, they are overexpressed [57]. On the other hand, MT-1/MT-2 are induced by a number of other stimuli, including glucocorticoids, stress conditions, ROS, cytokines, lipopolysaccharides or interferon, while MT-3 expression is not provoked by such stimuli [38,56]. However, according to the studies conducted on renal proximal tubule cells, it has been shown that MT-3 may be weakly and transiently induced by Zn or Cd ions [46,58]. MT-3 was discovered in the human brain and termed as a growth inhibitory factor because of its in vitro neuronal growth inhibitory abilities [59]. MT-3 is also known as a controller of metal ion homeostasis in the brain [2]. However, it is undeniable that MT is a very active molecule involved in many physiological processes, but at the same time, it is pathological, since it is suspected that it may play a role in the pathogenesis of AD. Its expression is apparently upregulated in regions of Aβ plaque in the pre-clinical [60] and clinical AD brain [61], as well as in the brains of transgenic AD mice: Tg2576, TgCRND8, and Tg-SwDI [62]. MT-1 and MT-2 are upregulated in response to injury; they protect against neuronal damage, neurotoxic insults, and ROS. They also regulate neuronal outgrowth as well as influence tissue architecture and cognition. MT-3 also protects against brain damage, antagonizes the neurotrophic and neurotoxic effects of Aβ, and influences neuronal regeneration [63]. However, MT-3 may play a more complex role in AD progress than other MTs. This protein isoform is characterized not only by metal-binding and ROS-scavenging properties like other MTs, but it also displays distinct protein-binding features not shared with other MT isoforms [2,64]. The protein-binding activity of MT-3 arises from its β-domain, while the α-domain is not directly involved [2,65]. Analysis of the MTs level in the brain of AD patients showed increased expression of MT-1 and MT-2 and decreased expression of MT-3 by 30% [25]. There are also contrary results showing that MT-3 expression is increased [66], or that a difference in the expression of MT-3 in AD was not noticed [67].

## 5. Metallothioneins and Metal Ions in the Context of Alzheimer’s Disease

As mentioned above, the high content of cysteine residues (about 30% of all amino acids) allows toxic metal detoxification and oxidative stress protection by MT as well as the maintaining of essential metals homeostasis including Zn and Cu as a metallochaperone [47]. The MT α-domain binds with the help of 11 cysteine residues, 4 Zn^2+^ ions, or 6 Cu^+^ ions. After filling places in this domain, metals bind to the β-domain and with the help of nine cysteine residues bind three Zn^2+^ or six Cu^+^ ions [47,68]. Besides that, these proteins bind monovalent metals from group 11 and divalent metals from group 12 with different geometry depending on the metal: As_6_-MT, Cd_7_- or Zn_7_-MT, or Ag_12_- or Cu_12_-MT, and Hg_18_-MT [55,68]. By coordinating metals to MT, the pKa of cysteine is reduced to six orders of magnitude. The result is the attachment of cysteine sulfur atoms to metals, which form thiolates. The metal–cysteine connection determines the secondary structure of the protein [68]. In combination with metals, MTs are more stable, as evidenced by the low dissociation constant value for MT clusters, which are complexes with metal ions coordinated by sulfur derived from cysteine residues [43,69].

As mentioned earlier, MTs show a prominent upregulation in the vicinity of the amyloid plaques. Presumably, such upregulation will benefit the neighboring cells considering the antioxidant, anti-inflammatory, and antiapoptotic properties of these proteins. Besides these features, MTs are also metal-binding proteins. It is a property of particular significance in AD, since Aβ plaques are enriched in zinc, copper, and iron ions, which are metals very likely to be involved in the aggregation of Aβ, and thus plaque formation as well as in the generation of ROS and neuroinflammation [4,25]. MTs bind Zn and Cu ions with high affinity, hence their ability to regulate the transport, storage, and inhibit the toxicity of those ions [55,68]. Almost all of the intracellular Zn(II) is bound to the metal-binding proteins, thereby limiting the amount of free Zn(II) in the cytoplasm [70]. MT-1/2 is a primary protein that bind Zn ions in the cells and when they are isolated from livers of various species they usually enclose seven Zn(II) ions [71]. It has been shown that thanks to the homeostatic buffering role of MTs and the possession of thiol groups, MT-1/2 can pass Zn to zinc enzymes and activate them directly or remove Zn from zinc-finger transcription factors and inactivate it. Those two isoforms can also indirectly affect processes dependent on supplying Zn by modulating Zn availability in the cells [72]. The transfers of Zn may have an important effect on cell differentiation, proliferation, and apoptosis, as well as on the regulation of gene expression [46,73,74]. Therefore, MTs play a crucial role as a donor and acceptor of metal ions [47]. According to the conducted studies, it was demonstrated that MT-2A in vitro decreases Aβ neurotoxicity of cultured cortical neurons probably because of a metal swap between Zn_7_-MT-2A and Cu(II)-Aβ [75]. A similar protective effect was previously demonstrated for Zn_7_MT-3 in in vitro studies [76]. Zn_7_MT-3 has a thiolate-disulfide couple which links zinc-thiolate cluster reactivity to Cu(II) reduction and removal from Aβ_1–40_-Cu(II) leading to the generation of oxygen-stable Cu(I)_4_Zn_4_MT-3 and redox-inert Aβ_1–40_-Zn(II). Taking into account the above, the existence of an underlying molecular mechanism has been revealed. The metal swap between Zn_7_MT-3 and soluble and aggregated Aβ_1–40_-Cu(II) abolished ROS production, which, in turn, explains the decreased cellular toxicity observed. In the recent in vivo research, the APP/PS1 mouse model, which is a double transgenic mouse with a chimeric mouse/human APP and a mutant human presenilin 1, was treated with sustained drug release of Zn_7_MT-3 administered straightway to the CNS. It showed that Zn_7_MT-3 can significantly improve cognitive deficits, ameliorate the morphology and function of the hippocampus, regulate metal homeostasis, abolish Aβ plaque load, and reduce oxidative stress and neuronal cell apoptosis in the transgenic mice. In addition, it has been confirmed that MT-3 can partially cross the BBB of AD mice. Therefore, Zn_7_MT-3 could be an effective AD suppressing agent and it has potential for applications in Alzheimer’s disease therapy [26]. In addition, recent studies on the metal-dependent interactions of the MT-3 β-domain with Aβ demonstrated that both Zn-MT-2 and Zn_3_-βMT-3 can decrease Cu^2+^-induced Aβ neurotoxicity, but only Zn_3_-βMT-3 has a specific affinity to Aβ. Through this interaction, a stable Zn-MT-3/Aβ complex is created which effectively prevents the formation of Cu-Aβ in high viscosity physiological fluids. This may be significant for explaining the function of MT-3 in AD neuropathology and for developing a therapeutic strategy for AD associated with MT-3 [2].

## 6. Transthyretin Functions and Its Role in Alzheimer’s Disease

Transthyretin (TTR), albumin, and thyroxine-binding globulin are three main proteins responsible for the distribution of thyroid hormones that are produced mainly in the liver. They are secreted into the blood and CSF [8,9,13] where they ensure proper distribution of hormones to tissues and maintain a pool of free hormones in the blood and CSF. TTR binds thyroid hormones (THs) in the form of L-3,5,5’-triiodothyronine (T3) and L-thyroxine (T4) and is considered the most important distributor of T4 in human blood [9]. TTR is coded by a single gene copy on the 18th chromosome in humans and is expressed in the liver, kidneys, pancreas, choroid plexus [77], retinal epithelium, and leptomeningeal epithelium [78]. TTR expression in the liver is regulated differently than in the choroid plexus. For example, the total level of transthyretin mRNA in the rat choroid plexus is 11.3 times higher than its level in the liver. Also, the activity of this protein in CSF differs from that in the liver [9]. 

TTR exists mainly as a tetramer composed of four identical subunits, consisting of 127 amino acid residues each, and its molecular weight is 55 kD [8]. The tetramer is a biologically active form of TTR, which can simultaneously carry two T4 molecules and it interacts with one molecule of retinol-binding protein (RBP), which is the carrier of vitamin A [79]. TTR has an important role as an intermediator in retinol transport. RBP is synthesized in the liver and its secretion into the blood is initiated by binding with retinol. TTR is combined in a complex with RBP before secretion into blood. The creation of the complex is designed to protect RBP against kidney glomerular filtration rate. Binding of RBP to TTR is considered as a positive regulation of the delivery of retinol by RBP from plasma to the liver cells [9]. TTR also binds apolipoprotein AI, lutein, norepinephrine oxidation products, and pharmacological compounds such as penicillin, salicylates, some non-steroidal anti-inflammatory drugs [80], and flavonoids [81].

Interestingly, the role of human TTR can be twofold. First of all, it is a protein from the amyloidogenic group, which occurs in pathological conditions and causes systemic amyloidosis. The tetrameric form of TTR can sometimes lose its stability, dissociating into a monomer, which can then misfold and form fibrils. This action causes senile systemic amyloidosis diseases in elderly people [82]. The T4 binding site has been described in the context of ensuring TTR stability. The presence of stable forms of TTR seems to be a factor that inhibits the formation of amyloid fibrils. Inhibition of fibril formation has been shown to occur when both T4 binding sites are occupied by inhibitors that bind to TTR [83]. The research carried out by Sato et al. [84] indicates that the chromium metal ions—Cr^3+^, contribute to the stabilization of the TTR tetramer. It has been hypothesized that Cr^3+^ causes an increase in T4 binding to TTR by electrostatic neutralization of the Glu54 TTR, which is topologically close to the T4-binding site. Increasing the binding of T4 to TTR would allow the higher thermodynamic stability and integrity of the TTR tetramer to be maintained, which in turn reduces the formation of amyloid fibers. In contrast to causing senile systemic amyloidosis diseases, TTR is involved in the neuroprotection of AD [13,85]. In favor of neuroprotection, it was shown that overexpression of wild-type tetrameric TTR in an APP23 transgenic mouse model of Alzheimer’s disease improved cognitive functions [86]. In the presence of TTR, Aβ aggregation decreases and its toxicity is abolished [54,87,88]. Moreover, TTR is one of the main proteins in human CSF binding Aβ [87,88]. Many studies over the years have indicated that an important aspect in the context of binding ability seems to be the structure of TTR, which is determined by the quaternary structure of the protein [89]. The NMR studies showed that the TTR binding site for Aβ contains amino acids around and inside the T4 binding site. Results of the aforementioned tests indicated shifts between the resonance signals of amino acids containing the T4 binding site, both in the presence of Aβ and in its absence, which confirms the participation of this binding pocket in Aβ binding [90]. The mechanism of inhibiting Aβ aggregation in vivo was also described [90]. It involves the binding of the hydrophobic region of the Aβ monomer through the TTR T4 binding site, which sequesters monomers that later form oligomers and abolish Aβ form β-sheet structure. The experiments also indicate that Aβ does not destabilize the TTR tetramer and that it has a greater influence on the inhibition of Aβ deposition. It suggests that the tetrameric form of TTR is probably the main element in the inhibition of Aβ aggregation [90]. This is confirmed by other in vivo studies which indicated that administration of TTR tetrameric stabilizers to AD transgenic mice improved the pathological condition and can improve the interaction between TTR and Aβ [91]. However, in contrast to that, there are hypotheses, confirmed also by recent studies, that the dissociation of the tetrameric form of TTR is necessary for the effective inhibition of Aβ cytotoxicity [85]. Some wild-type TTR tetramers dissociate into monomers, thereby acquiring the ability to bind small Aβ oligomers, thus preventing the formation of Aβ fibrils and their aggregation. In turn, another recent ThT fluorescence spectroscopy study shows that both forms of TTR—tetrameric and monomeric—can bind to Aβ oligomers reducing Aβ toxicity and fibril formation [92].

However, Aβ level depends not only on the method of APP proteolysis but also on the efficiency of the Aβ removal mechanism [8]. It was shown that TTR also takes part in the efflux of brain Aβ and peripheral clearance [54]. The study suggests that TTR participates in the catabolism of this protein because its interaction with Aβ contributes to both maintaining soluble Aβ at an appropriate level in CSF under homeostatic conditions and to removing deposited Aβ in amyloid plaques in the case of balance disorder and disease [3]. Recently, Cao et al. proposed that tetrameric TTR prevents Aβ cytotoxicity by sequestering and promoting the clearance of Aβ monomers while the dissociated form of TTR associates with Aβ oligomers to form non-toxic clusters that are more prone to digestion and removal [85]. In vivo and tissue culture studies revealed that in a murine model of Aβ deposition, TTR was overexpressed, probably due to an increase in the neuronal synthesis of TTR and interplay between TTR and Aβ, which decreased Aβ concentration, aggregation capacity, and toxicity in the context of the neuron and its environment.

The ability of TTR to reduce toxicity caused by Aβ oligomers was also observed when caspase-3 activity was measured. It is relevant for normal brain development and frequently activated death protease. SH-SY5Y neuroblastoma cells, used as a neuronal function and differentiation model, were incubated with TTR pre-incubated with Aβ_1–42_, TTR alone, or Aβ alone. Cells incubated with TTR alone and with TTR pre-incubated with Aβ_1–42_ showed caspase-3 activity at a similar level to control, non-treated cells. On the other hand, the cells treated only by Aβ presented an increase in caspase-3 activity [8]. Transmission electron microscope studies indicated that recombinant human TTR (huTTR), mouse TTR (muTTR), and human monomeric TTR (M-TTR) incubated with Aβ_1–40_ inhibited the formation of Aβ fibrils [87]. This protein has also metalloprotease function [13,93]. In vitro studies have shown that the presence of metal ions such as Fe^2+^ or Cu^2+^ has an impact on the conformational change of the TTR. Under the conformational changes, there is a modification of the dimer–dimer interface, rearrangement of residues related to Aβ neutralization, and TTR acquires Zn^2+^ binding ability and proteolytic properties [93,94,95]. To its natural substrates Aβ, apolipoprotein AI and neuropeptide Y can be included [9]. It is believed that this cryptic protease activity of TTR is used for cleavage of Aβ into shorter, non-amyloidogenic fragments and degradation of aggregated forms of Aβ peptide [63,96,97]. The capture of the Aβ protein may prevent amyloidosis, and disorder of this process can lead to the development of AD. The results of recent studies performed by Ciccone et al. indicate that the binding affinity of TTR for Aβ_1–28_ additionally increases from the micromolar to nanomolar range in the presence of copper ions [95], which indicates that copper plays an active role in the stabilization of TTR-Cu-Aβ complexes. The neuroprotective activity in TTR structural feature has also been observed in another development, more precisely in the wild-type TTR. It has the ability to participate in proteostatic processes, thus complementing the endogenous proteostatic apparatus by decreasing the concentration of aggregation-prone protein in a chaperone-like manner [98]. The interaction between TTR and Aβ is a heterotypic interplay, thus contributing to the maintenance of cellular protein homeostasis [99]. Therefore, TTR may be useful in preventing or treating amyloidosis [9,13].

## 7. Complexes of Transthyretin with Metallothionein in Alzheimer’s Disease

As mentioned above, TTR is considered a molecule performing a protective role in the course of AD due to its proteolytic activity. TTR may cause hydrolysis of Aβ, resulting in the formation of short peptides with less activity of amyloid plaques creation [9,54].

The formation of complexes between TTR and MT was observed [63,74]. The interaction between TTR and MT-1/2 was revealed by the use of a yeast two-hybrid system method [79]. This interaction was confirmed in vitro by co-immunoprecipitation, crosslinking, Western blot, and competitive binding. It was shown that the affinity of TTR to MT-1/2 is relatively high. The dissociation constant for the complex is Kd = 244.8 ± 44.1 nM, which is a comparable result with respect to other TTR ligands (e.g., Kd [TTR-RBP] = 800 nM, and Kd [TTR-T4] = 10 nM). Using a competitive binding method, it was found that the only cysteine residue (Cys10) of the TTR molecule is not involved in the interaction between TTR and MT, so it is not an interaction with a disulfide bridge formation. Moreover, using Western blot, the interaction of MT-TTR was confirmed once again, by detecting complexes in proteins isolated from the kidney and choroid plexus of the rat. Additionally, the addition of anti-MT and anti-TTR to the sample confirmed the presence of the MT-TTR complex, in which differences in electrophoretic mobility between the MT-TTR monomer and dimer were observed. It suggested that MT only binds TTR monomers, while the binding does not occur in the case of TTR dimers or tetramers. Colocalization of these proteins in the cytoplasm of the choroid plexus cells was demonstrated in vivo. Moreover, the presence of MT-1/2 in the endoplasmic reticulum suggests that such colocalization takes place in that compartment. It should be taken into account that the simultaneous presence of these two proteins in the endoplasmic reticulum and the fact that they form complexes in the CSF suggest that MT-TTR complexes can be located in the choroid plexus cells, or may be formed outside the cell, in the CSF. In addition, MT is present on both the inside and outside of the cell, which further confirms the possibility of interaction between these proteins [79]. Both in vitro and in vivo experiments indicate the existence of interactions between MT-1/2 and TTR.

Due to the fact that both TTR and MT affect metabolism and deposition of Aβ, the possibility for the interaction of TTR with MT-3 was also examined. Analysis using the yeast hybrid system showed the formation of MT-3-TTR complexes. The dissociation constant for this complex was 373.7 ± 60.2 nM. The interaction was confirmed by co-immunoprecipitation in the specific antibodies. In the case of TTR, precipitation was applied. Precipitation of MT-3 confirmed the interaction between these two proteins. Moreover, their co-localization was also present in the cytoplasm (as in the case of MT-1/2), in particular in the endoplasmic reticulum [63].

**Figure 4:**
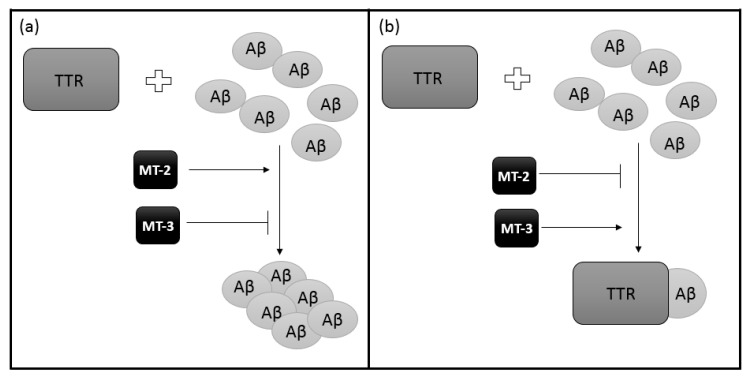
A schematic effect of different MT isoforms on TTR ability of Aβ binding: (**a**) MT-2 reduces binding interaction between TTR and Aβ so the removal of Aβ will be less efficient while (**b**) MT-3 increases the ability of TTR to bind Aβ, producing the opposite effect to MT-2.

Then, by competitive binding, the impact of the existence of MT-TTR interactions on the binding of Aβ by TTR was examined. Preincubation of TTR with MT-2 reduces the ability to bind Aβ with TTR (Figure 4a). Preincubation of TTR with MT-3 produces the opposite effect (Figure 4b). The presence of MT-2 or MT-3 without prior preincubation with TTR does not affect the binding of TTR-Aβ [63]. It has been shown that different isoforms of MT have different specificity of action. Thus, it can be concluded that with decreased expression of MT-3 and increased MT-2, the removal of Aβ will be less efficient, thus causing AD. In addition, it is believed that MT-3 reduces the harmful action of neurotoxic Aβ and the formation of toxic aggregates. This effect is probably related to the interaction of MT-3 with TTR [63].

On the basis of these results, it can be concluded that MTs have a great influence on the course of AD. MT is involved in the homeostasis of metal ions, in particular Zn, which is also involved in the pathology of AD. Besides Zn, metals such as Cu and Fe also react with Aβ and cause its aggregation and precipitation, thus resulting in the increased concentration of Aβ in the plaques [99,100]. In vitro and in vivo studies showed a mild effect of reducing the harmful effects of Aβ with MT-3. The possible reason could be the metal exchange between Zn_7_MT-3 and soluble or aggregated Cu^2+^-Aβ, leading to the suppression of ROS caused by the Cu^2+^Aβ redox cycle. On the other hand, it was found that the oxidation of cysteines in MT-3 can cause the release of Zn^2+^ and the aggregation of Aβ [99]. In the case of MT-1/2, no inhibition of Aβ was observed. In addition, the lack of MT-1/2 prevented the formation of plaques in the hippocampus and cortex. Accordingly, the exogenous injection of Zn_7_MT-2 into mice led to an increase in plaques in the hippocampus. These results indicate that the control of endogenous MT production may serve as a therapeutic agent [99,100].

Additionally, the discovery of the interaction between TTR and MTs that are present in the CSF and choroid plexus and are involved in the modulation of Aβ level underscores the importance of this interaction and its role in Aβ deposition and development of AD. In addition, the binding of MT-3 to the TTR monomer may cause disturbances in the formation of amyloid, being the main cause of systemic amyloidosis and familial amyloid polyneuropathy, which involves mutated variants of TTR [79]. The presence of MT-3 enhances the interaction of TTR with Aβ, resulting in increased degradation of aggregated forms of Aβ [63].

AD is a multifactorial disease with the lack of an effective treatment for years. Therefore, it is worth looking for further therapeutic targets and ligands that can be used in the future. There are many lines of research on AD, one of which is a treatment based on metal ions, metallodrugs, or chelating agents. Reports presented above suggest that MT-TTR complexes may be important not only for maintaining the body’s homeostasis but also for the deposition of Aβ in Alzheimer’s disease. The MT-3 fits perfectly into the assumptions of MTDLs and MPACs and in the future may become a new therapeutic agent.

## Abbreviations

Aβ	β-amyloid
AD	Alzheimer’s disease
CSF	Cerebrospinal fluid
CNS	Central nervous system
MT	Metallothionein
TTR	Transthyretin
APP	Amyloid precursor protein
NFTs	Intracellular neurofibrillary tangles
BBB	Blood–brain barrier
ROS	Reactive oxygen species
THs	Thyroid hormones
T3	L-3,5,5’-triiodothyronine
T4	L-thyroxine
RBP	Retinol-binding protein
PS1	Presenilin 1
